# Discrepancy of target sites between clinician and cytopathological reports in head neck fine needle aspiration: Did I miss the target or did the clinician mistake the organ site?

**DOI:** 10.1002/cam4.489

**Published:** 2015-06-24

**Authors:** Mahsa Khanlari, Yahya Daneshbod, Hanieh Shaterzadeh Yazdi, Sadegh Shirian, Shahrzad Negahban, Azita Aledavood, Ahmad Oryan, Bijan Khademi, Khosrow Daneshbod, Andrew Field

**Affiliations:** 1 Department of Cytopathology, Dr. Daneshbod Pathology LaboratoryShiraz, Iran; 2 Department of Pathology, School of Veterinary Medicine, Shiraz UniversityShiraz, Iran; 3 Department of Pathology, Shahrekord University, School of Veterinary MedicineShahrekord, Iran; 4 Shefa Neuroscience Research Center, Khatam-Al-Anbia HospitalTehran, Iran; 5 Brain and Spinal Cord Injury Research Center, Tehran University of Medical SciencesTehran, Iran; 6 Department of Otolaryngology, Shiraz University of Medical SciencesShiraz, Iran; 7 Hospital and University of Notre Dame Medical School SydneyFremantle, Australia

**Keywords:** Cytology, discrepancy, FNAC, head and neck, label error, target organ

## Abstract

The diagnostic accuracy of fine needle aspiration cytology (FNAC) of head and neck lesions is relatively high, but cytologic interpretation might be confusing if the sample is lacking typical cytologic features according to labeled site by physician. These errors may have an impact on pathology search engines, healthcare costs or even adverse outcomes. The cytology archive database of multiple institutions in southern Iran and Australia covering the period 2001–2011, were searched using keywords: salivary gland, head, neck, FNAC, and cytology. All the extracted reports were reviewed. The reports which showed discordance between the clinician's impression of the organ involved and subsequent fine needle biopsy request, and the eventual cytological diagnosis were selected. The cytological diagnosis was confirmed by histology or cell block, with assistance from imaging, clinical outcome, physical examination, molecular studies, or microbiological culture. The total number of 10,200 head and neck superficial FNAC were included in the study, from which 48 cases showed discordance between the clinicians request and the actual site of pathology. Apart from the histopathology, the imaging, clinical history, physical examination, immunohistochemical study, microbiologic culture and molecular testing helped to finalize the target organ of pathology in 23, 6, 7, 8, 2, and 1 cases respectively. The commonest discrepancies were for FNAC of “salivary gland” [total: 20 with actual final pathology in: bone (7), soft tissue (5), lymph node (3), odontogenic (3) and skin (2)], “lymph node” [total: 12 with final pathology in: soft tissue (3), skin (3), bone (1) and brain (1)], “soft tissue” [total: 11 with final pathology in: bone (5), skin (2), salivary gland (1), and ocular region (1)] and “skin” [total: 5 with final pathology in: lymph node (2), bone (1), soft tissue (1) and salivary gland (1)]. The primary physician requesting FNAC of head and neck lesions are incorrect in their clinical impression of the actual site in nearly 0.5 percent of cases, due to the overlapping clinical and imaging findings or possibly due to inadequate history taking or physical examination.

## Introduction

The head and neck region includes skin, bone, salivary glands, thyroid, soft tissue, and lymph nodes. All of which are subject to neoplastic and nonneoplastic changes 1. Cytopathologists are requested by clinicians to sample a particular site or organ, but on some occasions the cytological findings are not compatible with the clinicians target organ. Has the cytopathologist or interventional radiologist missed the site requested by the clinician or did the clinician recognize the organ involved incorrectly? Despite in clinical and pathology textbooks and some single reports have been mentioned that some organs such as: lymph node or salivary gland may be difficult to tell apart by clinical evaluation alone, there are no study focused on the discordance between the requesting physician's target organ and the final fine needle aspiration cytology (FNAC) diagnosis 1–9. The rate of this discordance will vary with the expertize of the clinician's patient examination, the utilized imaging and the skill of the practitioner carrying out the FNAC. These errors may have an impact on pathology search engines, healthcare costs or even adverse outcomes. The discordance rate will have a diagnostic and subsequently management impact on assessment of FNAC series, for example, FNAC of “salivary gland lesions” may yield parotid primaries, metastatic periparotid lymph nodes, skin or bone primaries which may have different management and surgical approaches.

## Materials and Methods

The cytology archive database of multiple institutions in southern Iran and Australia covering the period (2001–2011) were searched using keywords: salivary gland, head, neck, FNAC, and cytology. The total number of 10,200 head and neck superficial FNAC were included in the study from which 48 cases showed discordance between the clinicians request and the actual site of pathology. All FNAC were done under the guidance of sonography by cytopathologists. All the extracted reports were reviewed. The reports which showed discordance between the clinician's impression of the organ involved in FNAC request form, and the eventual cytological diagnosis were selected from total controversial reports. Metastatic tumor in a lymph node was considered discordant when the primary tumor site was outside the head and neck or in intracranial regions. Further investigations performed by the cytopathologist and physician to make the final diagnosis, were reviewed in available records. The cytological diagnosis was confirmed by surgical biopsy, cell block, and immunostaining, with assistance of imaging, clinical outcome, physical examination, molecular studies, or microbiological cultures.

## Results

The hospital centers were head and neck surgical referral centers from different cities from Iran and Sydney, Australia. The discordant cases were almost evenly distributed in these centers and mainly by general practitioners. Ear, Nose, and Throat (ENT) physicians do better than general surgeons or general practitioners.

### Clinical data

The data base search revealed 48 cases showing discordance between the clinicians request and the actual site of the pathologydemonstrated in FNAC.

Patients had an age range of 1–72 years. Clinical data, including the organ site on which the clinician requested the FNAC, along with the cytological and surgical pathology diagnoses and the procedure that assisted with the diagnosis such as the cell block, immunohistochemistry (IHC), molecular, microbiological culture, imaging, history, and physical examination, are presented in Table1.

**Table 1:** Clinicopathologic data of 48 controversial FNACs with histologic diagnoses and complementary diagnostic modalities needed to confirm origin and diagnosis

Case number	Age/sex	Requested organ	Cytology organ diagnosis	Histology	Complementary diagnostic modality
1 2	70/F	Salivary gland	Salivary gland tumor	Osteosarcoma	Imaging^1^
2	22/F	Salivary gland	Salivary gland tumor	Mandibular osteosarcoma	Imaging
3	44/F	Salivary gland	Salivary gland tumor	Soft-tissue inflammation	
4 3	60/F	Salivary gland	Salivary gland tumor	Chondrofibromyxoma	Imaging
5	45/M	Salivary gland	Salivary gland tumor	Ameloblatoma	Imaging
6 4	14/F	Salivary gland	Salivary gland tumor	Spindle cell rhabdomyosarcoma	IHC
7	17/F	Salivary gland	Salivary gland tumor	Bone myxoma	Imaging
8	23/M	Salivary gland	Epidermoid cyst	OKC	
9	17/M	Salivary gland	Squamous cell carcinoma	Epithelioma of Malherbe	
10	12/M	Salivary gland	Lymph node	Lymphoma	IHC
11	22/F	Salivary gland	Lymph node	T cell Lymphoma	History and IHC^2^
12	46/F	Salivary gland	Lymph node	Systemic lymphoma^3^	IHC, imaging and P/E
13	12/M	Salivary gland	Lymph node	Cellulitis	Culture and P/E
14	55/M	Salivary gland	Not sufficient for diagnosis	Lipoma	Imaging
15	48/F	Salivary gland	OKC	OKC	Imaging
16	1/M	Salivary gland	Ewing mandible	Ewing mandible	IHC and imaging
17 5	15/M	Salivary gland	Ganglioneuroblastoma	Ganglioneuroblastoma	IHC, history and Imaging
18	47/F	Salivary gland	Brown tumor	Brown tumor	History and Imaging
19 6	30/M	Salivary gland	Brown tumor	Brown tumor	History and Imaging
20	11/F	Salivary gland	NC	Osteopetrosis	Imaging
21 7	46/M	Lymph node	Cancer	Metastatic meningioma	Imaging
22	51/F	Lymph node	Granuloma	Spindle squamous cell carcinoma	IHC and history
23	2/F	Lymph node	Small round cell tumor	Mandibular neuroblastoma	IHC
24	46/F	Lymph node	Epidermoid cyst	Metastatic squamous cell carcinoma	P/E
25	42/M	Lymph node	Mixed tumor	Skin adnexal tumor	
26	32/F	Lymph node	Carotid body tumor	Carotid body tumor	Doppler sono + IHC
27	34/F	Lymph node	Neurofibroma	Neurofibroma	History and P/E
28	2/F	Lymph node	Neurofibroma	Neurofibroma	IHC and imaging
29	55/F	Lymph node	Salivary lymphoepithelial lesion	ND^4^	Imaging
30 8	50/F	Lymph node	Calcified material	Calcified Goitre	Imaging
31	15/M	Lymph node	NC	ND	Imaging (salivary stone seen in sialography)
32	66/F	Lymph node	Epidermal inclusion cyst	Epidermal inclusion cyst	P/E
33	66/M	Soft tissue	Skin	Basal Cell Carcinoma	History
34 15	35/M	Soft tissue	Cyst	Hydatid cyst	Imaging
35	64/F	Soft tissue	Salivary gland tumor	Mixed tumor	
36	55/F	Soft tissue	Chondroid tumor	Chondrosarcoma	Imaging
37	62/M	Soft tissue	Squamous cell carcinoma	Squamous cell carcinoma^5^	History^5^
38	6/F	Soft tissue	Histiocytosis	Histiocytosis of bone	Imaging and P/E
39	4/M	Temporal soft tissue	Histiocytosis	Histiocytosis of bone	Imaging
40	34/M	Soft tissue	NC	Fibrous dysplasia	Imaging
41	55/M	Soft tissue	NC	Fibrous dysplasia	Imaging
42	40/F	Soft tissue	Actinomycetoma	ND^4^	Imaging and Culture
43 9	71/F	Soft tissue	Multiple myeloma	ND^4^	Imaging and IHC
44	56/M	Skin	Salivary gland tumor	Adenoid cystic carcinoma	P/E, imaging
45	47/F	Skin	Soft-tissue inflammation	Soft-tissue fungal infection	Culture
46	27/F	Skin	Inflammatory process	Osteomyelitis	Imaging and culture
47 16	22/M	Skin	Lymph node	LLL	P/E
48 16	57/M	Skin	Lymph node	LLL	P/E, molecular (PCR)

FNAC, fine needle aspiration cytology; OKC, odontogenic keratocyst; IHC, immunohistochemistry; LLL, localized leishmania lymphadenitis; ND, not done; NC, noncontributory; P/E, physical examination; M, male; F, female.

1 Any radiology work up.

2 Previous lymphoma of breast.

3 Lymphoma with secondary involvement of Salivary gland lymph node.

4 Cell block only.

5 Previous squamous cell carcinoma (SCC) of esophagus.

### Requested organ

The clinicians requested fine needle aspiration biopsy (FNB) on the following target organs: salivary gland (20), lymph node (12), soft tissue (11), and skin (5). Besides histopathology, imaging, clinical history, physical examination, immunohistochemical studies, microbiologic culture, and molecular tests helped to finalize the target organ of pathology in 28, 6, 8, 10, 4, and 1 cases, respectively.

### Labelled organ versus final organ pathology mismatch

#### Salivary gland

**Figure 1:**
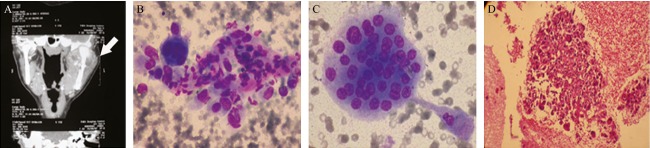
Imaging of this salivary-like mass confirmed bony origin (mandibular mass) (A, arrow), cytology showed spindle cells and multinucleated giant cells (B and C Wright, 200×), which by cell block showing malignant osteoid, osteosarcoma was proved (D) (hematoxylin eosin, 200×).

**Figure 2:**
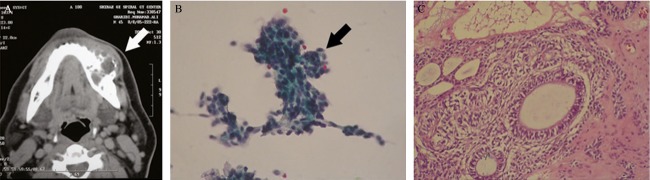
Imaging of this salivary-like mass confirmed bony origin (mandibular mass) (A, arrow), cytology showed spindle cells and diagnosed as mixed tumor (B, arrow Wright, 200×), which histology proved ameloblastoma (C) (hematoxylin eosin, 200×).

**Figure 3:**
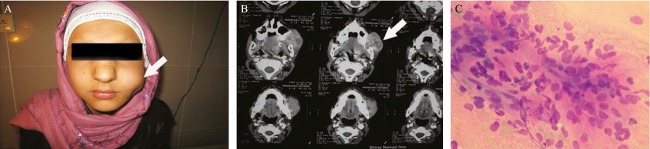
Clinical and imaging of this salivary-like soft-tissue mass (A, arrow) (B, arrow), which cytology showed spindle cells and diagnosed as mixed tumor (C Wright, 200×), histology, and immunohistochemistry proved showed Spindle cell rhabdomyosarcoma.

There were 20 FNAC requests for “salivary gland” FNA, including seven cases where an initial FNA diagnosis favored a salivary gland tumor, however, further excision, IHC and imaging study showed osteogenic sarcoma 2 (Cases 1 and 2) (Fig.1), soft-tissue inflammation (Case 3) chondrofibromyxoma 3 (Case 4), ameloblastoma (Case 5) (Fig.2), spindle cell rhabdomyosarcoma 4 (Case 6) (Fig.3) and bone myxoma (Case 7). Two cases were diagnosed on FNA as epidermoid cyst and squamous cell carcinoma but these were actually an odontogenic keratocyst and epithelioma of Malherbe respectively (Cases 8 and 9). Four cases were diagnosed as secondary lymphoma and cellulitis (Cases 10, 11, 12, and 13) (confirmed by IHC and culture). Six cases came out as: lipoma (Case 14), odontogenic keratocyst (Case 15), Ewing's sarcoma of mandible (Case 16), ganglioneuroblastoma 5 (Case 17), and two cases of brown tumor 6 (Cases 18 and 19) (confirmed by IHC, history, and imaging). In one of the 20 discrepant “salivary gland” cases, the cytological diagnosis was inconclusive but osteopetrosis was demonstrated by imaging and biopsy (Case 20).

#### Lymph node

**Figure 4:**
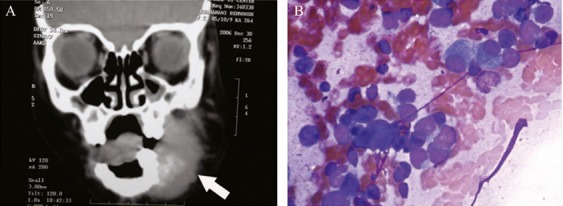
Imaging of this soft-tissue mass confirmed bony origin (mandibular mass) (A, arrow) Cytology showed round cell tumor and osteoblasts (B) immunohistochemistry proved neuroblastoma of mandible (Wright, 200×).

There were 12 FNA request for “lymph node” FNA. The cytological diagnosis of five cases were carcinoma, granuloma, small round cell tumor, epidermoid cyst, and pleomorphic adenoma and these cases were shown to be metastatic meningioma 7 (Case 21), spindle cell squamous cell carcinoma (Case 22), mandibular neuroblastoma (Case 23) (Fig.4), squamous cell carcinoma (Case 24), and skin adnexal mixed tumor (Case 25), respectively (confirmed by history, IHC, histology, imaging, and physical examination).

One case on cytology and histology was a carotid body tumor (Case 26) and two cases were mandibular neurofibromas (Cases 27 and 28) (physical examination, imaging and IHC helped confirm the diagnosis). The diagnoses on FNA in three cases were salivary lymphoepithelial lesion (Case 29), calcified material 8 (Case 30 and 31), and epidermal inclusion cyst (Case 32), interestingly all these four case histology was noncontributory, and diagnosis were confirmed on imaging and clinical examination.

#### Soft tissue

There were 11 requests for FNA on “soft tissue”. In two cases the lesions were a skin basal cell carcinoma (Case 33) and hydatid cyst (Case 34) based on cytology, surgical biopsy, and imaging. In another five cases the diagnoses were salivary gland pleomorphic adenoma (Case 35), bone chondrosarcoma (Case 36), metastatic squamous cell carcinoma (Case 37), and histiocytosis (Cases 38 and 39) based on FNA biopsy, imaging, IHC, history, and physical examination. In two cases, the FNA was noncontributory but the surgical diagnosis was fibrous dysplasia (Cases 40 and 41) supported by imaging. In two cases, the cytology diagnosis was actinomycetesoma (Case 42) and multiple myeloma 9 supported by microbiological culture, IHC, and imaging (Case 43).

#### Skin

There were five requests for FNA of “skin” and the final target diagnoses of three cases were adenoid cystic carcinoma (Case 44), soft-tissue fungal infection (Case 45) and osteomyelitis (Case 46) confirmed by physical examination, microbiological culture, imaging, and histology. The final two diagnoses were leishmania lymphadenitis supported by physical examination and molecular studies (Cases 47 and 48).

## Discussion

Head and neck lesions can be easily seen and palpated due to their superficial locations and are highly suitable targets for FNA as the initial diagnostic test because of its high sensitivity and specificity for both neoplastic and nonneoplastic lesions 10–13. Ultra sonographic-guided FNA of superficial organs is increasingly performed by with high accuracy to a variable extent depending on the site. However, understanding the complex anatomy, disease processes, and patterns of nodal spread in the head and neck make this technique ideal when applied with adequate clinical informations.

In our experience, physicians often request FNAC for any abnormal growth before they take thorough history, perform an adequate physical examination, diagnostic ultrasound or other imaging studies 14. Inadequate clinical evaluation may lead to requests for FNAC on the wrong organ. Clinicians may make a mistake in detecting accurate location of head and neck lesions due to variations in the presentation of the lesions, for example, the sites and range of pathology in major and minor salivary gland areas and their mimics in soft tissue and bone of these sites 12. All these errors may have adverse outcome for the patient including cost burden and surgical complications.

The clinical features of palpable mass lesions in the head and neck region overlap for skin, soft tissue, salivary gland, and bone or even with the frequency of different organ pathology (infectious and neoplastic processes). This very overlapping of clinical presentation makes the FNAC such an excellent minimally invasive first diagnostic test for head and neck lesions. Clinician's presenting history, physical examination, and imaging of head and neck growth need to be best correlated with the FNAC findings.

Providing a previous history of malignancy by the referring clinician will assist the pathologist in assessing lymph nodes and other palpable lesionsaccording to patterns of nodal spread in head and neck. Proper history was complementary in lymphoma involvement (Case 11), metastatic meningioma 7 and ganglioneuroblastoma 5 (Cases 21 and 17), uterine cervical squamous cell carcinoma metastasizing in neck as a “spindle cell SCC” incorrectly diagnosed as “granuloma” in FNA (Case 22) and skin tumor presenting as a recurrence (Case 33).

Thorough physical examination of skin lesions of histiocytosis or neurofibromatosis (cafe au lait spots) by cytopathologist can be clues to correctly diagnose a bone or neural lesions which were missed/not mentioned by referring clinician (cases 27 and 38).

Multifocal lesions on the face suggest a primary skin lesion rather than a pleomorphic adenoma (Case 32), although recurrent pleomorphic adenoma can be multifocal, or the previously undetected submandibular tumor in a patient with requested skin FNAC (Case 44), or in endemic areas for leishmaniasis the recognition of skin lesions that direct a FNAC of the localized leishmania lymphadenitis and its diagnosis (Cases 47–48).

Recently, cytopathologists have learned to use ultrasound machines to assist them in performing FNA procedures. Imaging particularly the more readily available and clinically flexible ultrasonography should be used as an ancillary tool for both clinicians and pathologists to significantly improve FNAs in smaller, nonpalpable lesions and target complex lesions to confirm both organs of origin as well as the diagnosis with confidence and accuracy, and achieving a better outcome. For example, mandibular tumors can be cytologically and histologically mistaken for salivary tumor 2,3 (Cases 1, 2). Odontogenic tumors with soft-tissue extension can be distinguished from skin or salivary lesions (Cases 5, 8).

FNA biopsy of bone lesions is a reliable diagnostic test for metastatic and primary bone tumors. Areas of difficulty were due to inadequate sampling or misclassification with regard to the exact site of malignancy (Cases 1, 2, 4, 7, 16, 18, 19, 20, 36, 38, 39, 40, 41, 43, 46). A bony mandibular lesion with an overlying suppurative sinus is suggestive of actinomycetes or osteomyelitis (Cases 42, 46).

Imaging also helps confirming a calcified goiter or salivary duct stones (Cases 30, 31). Color Doppler and immunostains on cell block helps in diagnosis of a hypocellular carotid body tumor (Case 26). Demonstration of bilaterality of salivary lesions and the absence of a true mass in imaging, helps to diagnosis lymphoepithelial disease rather than lymphadenitis (Case 29).

Cell blocks where available can be corroborated with immunophenotyping and immunocytochemistry as a method complementary to cytology in “tumor of origin/diagnostics” of lymphoma, round cell tumors, and spindle cell carcinomas 15,16 (Cases 6, 10, 11, 12, 22, 23, 26). And in the same way, molecular testing can be useful, for example, polymerase chain reaction (PCR) to confirm mycobacterial or, among our cases, leishmaniasis (Case 48).

In conclusion mislabeling of the target organ for a FNA requested by a clinician may be due to the overlapping clinical and imaging findings of head neck lesions but is exacerbated by an inadequate history taking or incomplete or poor physical examination by the clinician. The clinicians should provide us with all possible clinical and radiological information, it is also good practice to look for this information if the morphological findings do not fit the clinical suspicion, especially in the current era of electronic medical records or a phone call should suffice. Cytopathologists should be ready to seek clinical clues by directly questioning the patient and examining the patient as required, prior to performing the FNAC, or returning to ask questions of the patient after rapid on site assessment of the FNAC material. Imaging prior to the FNAC or at the time of the FNAC plays a crucial role in defining the site and organ involvement of the lesion. Microbiological cultures, immunocytochemical study of FNA, and cell block biopsy material and molecular methods are essential ancillary tests to confirm the diagnosis, when available.

FNAs of the Head and neck can be easily confused, not only because of the clinical similarity between lesions but also because of the overlap in cytomorphologic features of the aspirated cells. Although no one single cytomorphologic feature is diagnostic, a combination of clinical parameters noted earlier should raise the possibility of diagnosis.

Proper technique and recognition of these pitfalls, as well as simultaneous cytopathologist and clinician work ups are needed to achieve a successful FNA diagnosis and avoid discrepancy of target sites between clinician and cytopathological reports.

## Conflict of Interest

None declared.
